# A dataset for predicting cloud cover over Europe

**DOI:** 10.1038/s41597-024-03062-0

**Published:** 2024-02-27

**Authors:** Hanna Svennevik, Steven A. Hicks, Michael A. Riegler, Trude Storelvmo, Hugo L. Hammer

**Affiliations:** 1 University of Oslo, Department of Geosciences, 0315 Oslo, Norway; 2 Simula Metropolitan Center for Digital Engineering, Department of Holistic Systems, Pilestredet 52, 0167 Oslo, Norway; 3 OsloMet – Oslo Metropolitan University, Department of Computer Science, Pilestredet 52, 0167 Oslo, Norway

**Keywords:** Atmospheric dynamics, Scientific data, Computer science

## Abstract

Clouds are important factors when projecting future climate. Unfortunately, future cloud fractional cover (the portion of the sky covered by clouds) is associated with significant uncertainty, making climate projections difficult. In this paper, we present the European Cloud Cover dataset, which can be used to learn statistical relations between cloud cover and other environmental variables, to potentially improve future climate projections. The dataset was created using a novel technique called Area Weighting Regridding Scheme to map satellite observations to cloud fractional cover on the same grid as the other variables in the dataset. Baseline experiments using autoregressive models document that it is possible to use the dataset to predict cloud fractional cover.

## Background & Summary

Climate change is stated as one of the biggest challenges of our time, resulting in many unwanted effects. The UN Secretary-General António Guterres’ calls the latest IPCC Climate Report^1^ a ‘Code Red for Humanity’0^2^. Warmer global average temperatures will result in more frequent and more intense heat waves. It will also result in a higher evaporation rate, causing the atmosphere to hold more vapour and increasing the risk of flooding and other extreme weather phenomena. Glaciers and ice sheets will melt and ocean water will expand as it warms, raising the sea level^3,4^. Recently we have witnessed a range of extreme weather phenomena, and attribution science states that climate change is, at least partly, the cause^5^.

Cloud feedbacks, the response of clouds to a warmer climate, are associated with high levels of uncertainty^6^. In the current climate, clouds have a cooling effect^7^. Clouds interact with radiation, and it is expected that changes in cloud fraction cover (CFC) (the portion of the sky covered by clouds) will affect global warming, which will affect both climate and society, such as agriculture and solar energy production.

Numerical climate models (NCMs) solve differential equations of the physics driving the climate, including the atmosphere, oceans, land surface, and ice. NCMs are essential tools to predict future climate. Some variables can be projected well, such as temperature and pressure, while other variables, such as CFC, are associated with far more uncertainty^6^.

In this paper, we present the European Cloud Cover dataset (ECC), which can be used to potentially improve the projection of CFC in the future. The dataset consists of satellite observations of CFC and observations (reanalysis) of air temperature (T), surface pressure (SP), and specific and relative humidity (SRH). The main application of the dataset is as part of the following two-step statistical procedure^8^. First, use the ECC dataset to train a model that can predict CFC from T, SP, and SRH. Secondly, given projections of T, SP, and SRH from an NCM, insert these projections into the trained model from the previous step to project future CFC. The input variables used in such a procedure must satisfy that 1) historical observations are available of good quality and 2) are reliably projected by NCMs. The environmental variables T, SP and SRH where chosen since they satisfy these two requirements.

Given the high spatial and temporal resolution in the ECC dataset (latitude × longitude × time × number of variables = 81 ×161 × 129312 × 5) and the complexities of cloud formations, we believe that machine learning (ML) can be useful to predict CFC from other variables. To the best of our knowledge, this is the first dataset created for using ML to predict cloud formations. ML, especially deep learning (DL), has been significantly less used in climate research compared to other related problems like weather forecasting^9,10^. We believe that the ECC dataset can increase the interest in using ML and DL within cloud physics and climate research.

A challenge with our suggested observation-based approach for projecting future CFC using the ECC dataset, is that the formation of clouds are also driven by processes that are not captured by the environmental variables used in the ECC dataset. For example, many important physical processes are happening on a smaller scale. Recently NCMs are developed that integrate advanced cloud physics models as part of the climate model, e.g.^11^. However a substantial challenge is that these models are computationally too expansive to be able make climate projections decades into the future^12^. Recently attempts have been made to train ML and DL models to replicate the main properties of advances cloud physics models, but where the simulations are computationally less demanding. The approach are based on running the cloud physics models for some time period, say two years, and learn a ML or DL model to replicate the properties of the simulated data, see e.g.^12,13^. The approaches are promising, but are still not able to run on fully realistic scenarios and rather resort to simpler earth models such as the whole earth covered ocean (aqua-planet).

Overall, an observational-based approach, represented by the ECC dataset, and a simulation based approach using cloud physics models simulations and ML, represent two different data driven approaches to improve cloud parameterization in NCMs. A potential interesting application of the ECC dataset, could be to develop ML methods that use both sources of data.

The main contributions of this paper are as following: (i) We present a publicly available dataset that can be used to predict CFC, (ii) we present the Area Weighting Regridding Scheme (AWRS), a new technique to bring satellite observations and the other observations on the same geographical grid, (iii) we present a set of baseline experiments using autoregressive regression models that aim to show how the dataset can be used to predict CFC from T, SP, and SRH and (iv) we outline future applications and interesting research questions using the dataset.

## Methods

The ECC dataset consists of hourly data on a 0.25° uniform grid resolution from April 2004 to December 2018 and is comprised of the five variables; T, SP, SRH, and CFC. T and SP were chosen because they are reliable and fundamental meteorological variables. SRH because they are essential in cloud formation. The variables were all retrieved from the surface or the closest pressure level (1000hPa).

The ECC dataset used two sources of data. The first source is the 5th Generation Reanalysis data (ERA5) from the European Centre for Medium-Range Weather Forecasts^14^. Reanalysis is as close to observations as one can get while still obtaining complete and coherent data in both space and time. It is produced using a forecast model to assimilate observations. Data assimilation takes observations as input and tries to make an accurate estimate of the state of the system that is as consistent as possible with the available observations at all times. This includes observations retrieved from satellites, ships, buoys, airplanes, and ground-based stations. There are multiple global reanalysis datasets available, and they are all different. It depends on the forecast assimilation system used and observations assimilated^15^. ERA5 was elected because of its fine resolution and recent release date of January 2020.

The second source is METeosat Second Generation (MSG) cloud mask from the European Organisation for the Exploitation of Meteorological Satellites (EUMETSAT)^16^. Several candidate satellites were considered before arriving at the combination of datasets presented in this paper. Spatiotemporal consistency and resolution were given top priority to provide the best dataset to train models that can predict CFC from the other variables. The variable cloud mask is provided by many satellites, bringing valuable information in itself, but also for the retrieval of other variables restricted to cloud-free conditions, such as humidity. The MSG satellite is in a geostationary orbit and has an exceptional temporal resolution, with scans every 15 minutes. Knowing that the average lifetime of a cloud is approximately 60 minutes or less, the selected data set was found to be the most suitable for the purpose of the dataset^17^.

**Fig. 1:**
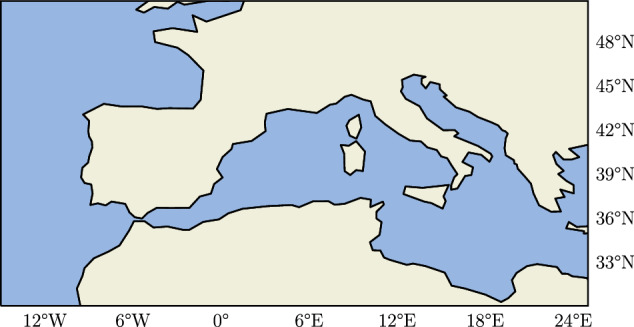
Domain of the ECC dataset.

The geographical domain is between 30° and 50° latitude (degrees north), and −15° and 25° longitude (degrees east) covering Central Europe and North Africa as shown in Fig. 1. The dimensions of the grid is 81 × 161 pixels.

Precipitation formation and cloud optical thickness (the degree to which the cloud prevents light from passing through) is affected by changes on a microphysical level (particles forming a cloud). However, they are undeniably closely related to the macrophysical (unit) properties of clouds as well. Imagine precipitation without a cloud fractional cover. Reliable estimates of large-scale variables are available from using reanalyses or other climate models. Restricting the focus here to the macrophysical aspects of clouds makes it reasonable to choose these as features, thereby ensuring that it is possible to build usable applications of projecting future CFC from NCM as described in the Introduction.

### Area Weighting Regridding Scheme (AWRS)

This section provides a detailed description of the necessary data processing done for the compilation of ECC, transforming clouds masks provided in *space-view* to cloud fractions on a uniform grid. EUMETSAT doesn not provide suitable software to tackle this particular task (personal communication with EUMETSAT staff). Therefore, to build the dataset, we developed a new regridding scheme, named “Area Weighting Regridding Scheme” (AWRS). Common schemes for solving similar tasks are mean, nearest neighbor or area weighting. We chose an area-weighted scheme since the distance between the satellite observations varied, and therefore the areas associated with each cloud satellite observation.

**Fig. 2:**
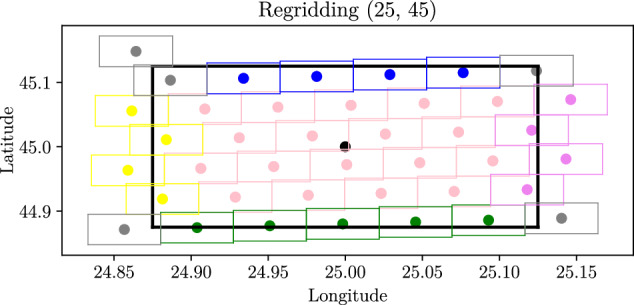
Example showing the contributing pixels to the remapping of pixel (**25,45**). The pixels from the satellite are classified into corner (grey), center (pink), right (purple), left (yellow), lower (green), and upper (blue) boundary. The dense black line is the pixel in gridECC, and the other pixels show the contributing pixels from gridMSG.

Let the subscripts denote the dataset pertaining to a particular grid, gridMSG refers to the space-view grid of the MSG and gridECC refers to the uniform grid originating from ERA5. GridECC is identical to gridERA5. Figure 2 shows a section of the real data, and gives an overview of the regridding problem. The filled circular points in gray, pink, purple, yellow, green and blue, show the geographic locations where MSG cloud information is available. The cloud information in these locations is available as zero or one for every time point, where one denotes that clouds were present and zero that clouds were not present. The dense black rectangle shows a pixel in gridECC, and the aim of the regridding method is to compute the CFC in each ECC pixel from the MSG cloud information. A challenge is that the horizontal and vertical distances between the circular points are not constant, but are increasing with increasing distance from nadir point. This means that a circular point with larger distances to its neighbors should contribute more to the CFC in the ECC pixel compared to a circular point with shorter distances to its neighbors. However, since we only know the cloud information in the circular points, the level of contribution relative to each other is not known. One natural solution is to compute a partition of the geographic area with one satellite observation in each part of the partition. The parts would then be trapezoid shapes with the longest side away from the nadir point. The trapezoid shapes are on the spherical earth surface, and to the best of our knowledge an analytic solution for the area of a trapezoid shape on a spherical surface does not exists. However, for a rectangle an analytic solution exists, and therefore we approximated the trapezoid shapes to rectangles. As shown in Fig. 2, the distances between the satellite observations did not change much, and the rectangle approximation was good, i.e. the size of a computed rectangle represented well how much the associated satellite observation within the rectangle contributed to the computed CFC for the associated ECC rectangle.

The CFC for a given pixel in GridECC, at some given time point, is computed as the area weighted average over the cloud information for each rectangle. More specifically, let *N* refer to the number of MSG rectangles that are within or partly within the ECC rectangle, and let ${m}_{i},i\in 1,2,\ldots ,N$ refer to if there were cloud or not in the circular point within rectangle *i*. Further, let ${a}_{i},i\in 1,2,\ldots ,N$ refer to the area of the part of the MSG rectangle *i* that is within the ECC rectangle. The area weighted CFC are then computed as the weighed average

1 $$CF{C}_{ECC}=\frac{1}{A}\mathop{\sum }\limits_{i=0}^{N}{a}_{i}{m}_{i}$$

where *A* refers to the total area $A={\sum }_{i=0}^{N}{a}_{i}$. For the sake of simplicity, the corner pixels, shown in gray in Fig. 2, were omitted from the calculations of cloud fraction.

### Missing Data

**Fig. 3:**
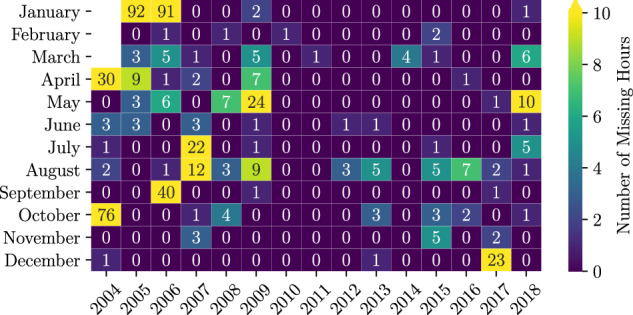
A heatmap summarising missing hours per month for all years.

**Fig. 4:**
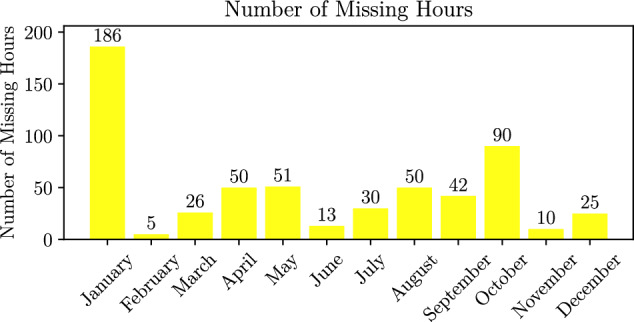
A barplot showing the monthly sum of missing values. This excludes the contribution from the period of 2004 before the satellite was operational.

Missing values are inevitable when working with observational data. Sensors occasionally fail to collect measurements, resulting in loss of data. This can either be individual pixels or entire disks. Contributing NaN pixels are counted and stored for future use in ECC. Missing timesteps result in missing disks. When available, the closest time step within the previous and trailing 45 minutes was used to fill the gap. A summary of missing timesteps per month in the dataset is provided in Fig. 3. Aggregation of missing timesteps per month is presented in Fig. 4. The plot is meant to illustrate any seasonal biases. The months of 2004 prior to the time at which the satellite became operational are not included in the statistics of missing values.

METeosat provides a two-satellite system, and occasionally both the standby and the operational sensors scan at the same time. In cases of technical failures, the standby scan is used. The scans are done from a different nominal position. However, the coordinate systems remain the same since the standby scan is rectified to the position of the operational satellite before the product is released (personal communication EUMETSAT staff). Comparing simultaneous measurements for the operational and the standby METeosat satellites, it becomes clear that they are dissimilar. However, this does not occur very frequently, and we have thus not attempted to quantify the magnitude of the parallax to correct for the bias this may introduce.

## Data Records

The ECC dataset and the code to create the dataset are available from the Open Science Framework^18^. An overview of the data records in the ECC dataset is shown in Table 1. The dataset is divided into files where each file consists of data for one variable over one month. The files are in the Network Common Data Form. The file naming convention is 20YY_MM_X.nc, where YY, MM and X, refer to year, month and type of variable, respectively. With X equal to q, r, sp, t2m and tcc, the file consists of measurements of specific humidity, relative humidity, air pressure, temperature two meters above surface level and cloud fractional cover, respectively. The variable tcc is available from April 2004 to December 2018, while the other variables are available from January 2004 to December 2018. The dataset thus consists of 297 files in total.

**Table 1:** Overview of the data records in ECC dataset.

Property	Quantity
Geographic area (degrees)	(**30,50**) north, (−15,25) east
Geographic resolution (degrees)	0.25
Grid size (latitide × longitude)	(81 × 161)
Recording time span	April 2004–December 2018
Temporal resolution	Hourly

**Fig. 5:**
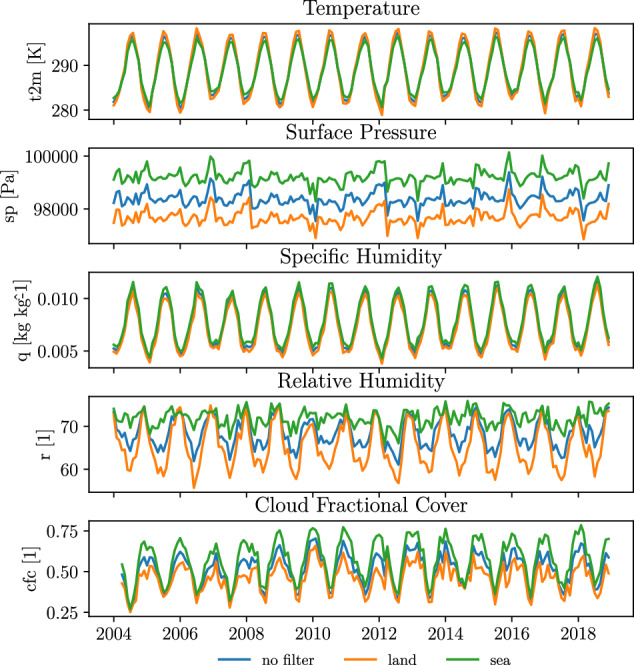
Spatially averaged monthly values. Filters are applied for land and sea.

Figure 5 shows the spatially averaged monthly mean values for all variables over the period with collected data. Seasonal effects and differences between land and sea are evident among all variables. For temperature and relative humidity, a more pronounced seasonal cycle over land, compared to the sea, is evident. The remaining variables appear to have a small shift towards higher values over the sea. This is expected because the pressure decrease with altitude, the sea has a high heat capacity (ability to retain heat), and is a source of humidity.

**Fig. 6:**
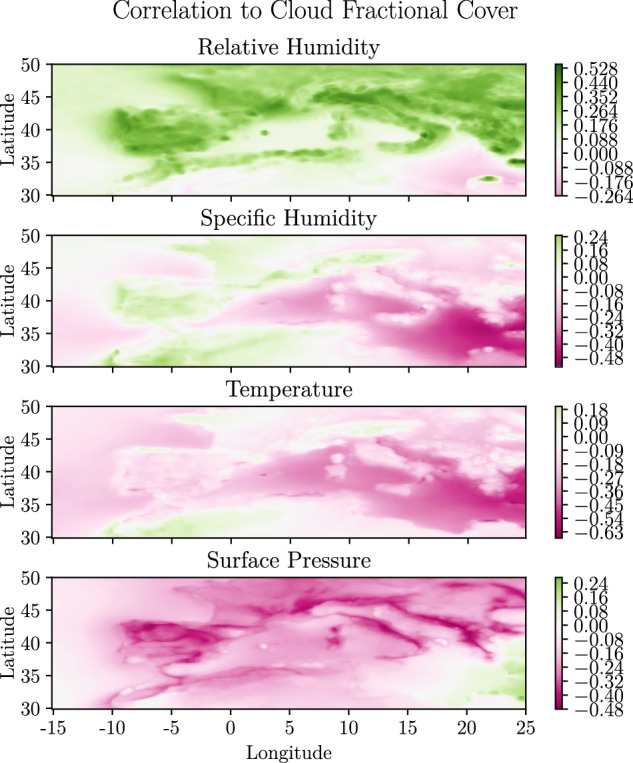
Contour plot showing the correlation coefficient between environmental variables and cloud fractional cover.

The linear correlation coefficients from pairs of cloud cover and the environmental variables T, P, SRH are shown in Fig. 6. Correlation describes how strongly a pair of variables are linearly related. A positive (negative) correlation represents a positive (negative) association between variables. Overall, we observe fairly strong correlations between the environmental variables and cloud fractional cover, indicating that it should be possible to predict cloud fractional cover from the environmental variables. However, note that there are strong spatial variations in the correlations and especially between land and sea. Over land relative humidity is dominated by a positive correlation with cloud cover, which means that higher relative humidity is associated with more clouds. However, in some parts of Africa and the sea in the eastern Mediterranean, the correlation is negative. The image of the surface pressure is remarkably similar, showing a similar pattern but with opposite signs. The negative sign seems reasonable since high pressure is often associated with sinking motions in the atmosphere and clouds are formed by rising motions. Specific humidity shows a clear shift at longitudinal degree 10. The land area in the west shows a positive correlation with cloud cover, while the eastern part shows a negative correlation. In most locations, the temperature is negatively correlated with cloud cover except in parts of the Alps, the north coast of France, and North Africa. This seems reasonable since warmer air can retain more vapour.

## Technical Validation

We first evaluated the validity of the developed AWRS method, by comparing it with the Climate Data Operator (CDO)^19^. CDO provides functionality to compute grid areas of a uniform grid. We verified that our AWRS and the CDO methods produced the same results on a uniform grid.

However, in contrast to our developed AWRS method, CDO cannot be used on a non-uniform grid, i.e., that the different grid cells are of different sizes, which was required to produce the ECC dataset.

We further evaluated the validity of the dataset, by testing if it was possible to use the dataset to predict CFC from the environmental variables. We trained autoregressive multiple regression models for each geographic grid cell to predict CFC from the environmental variables;

2 $$CF{C}_{t}={\beta }_{0}+{\beta }_{1}{T}_{t}+{\beta }_{2}S{P}_{t}+{\beta }_{3}R{H}_{t}+{\beta }_{4}S{H}_{t}+\mathop{\sum }\limits_{h=1}^{L}{\alpha }_{h}CF{C}_{t-h}+{\varepsilon }_{t}$$

where $CF{C}_{t},{T}_{t},S{P}_{t},R{H}_{t}$ and *SH*_*t*_ refer to cloud fractional cover, temperature, surface pressure and relative and specific humidity at time *t*, respectively. A separated model was trained in each geographic grid cell due to the strong variations in the dependency between the variables spatially, as shown in Fig. 6. The time period 2004 to 2013 was used to estimate the unknown parameters in the model (the *β*’s and *α*’s). The period 2014 to 2018 was used to evaluate the models’ ability to predict CFC one hour into the future. Prediction error was measured using mean absolute error (MAE).

The results are shown in Table 2. The results show that it is possible to predict CFC from the environmental variables with a fairly small error. We further see that if the autoregressive part or the environmental variables are removed from the model (i.e. setting ${\beta }_{1}=\cdots ={\beta }_{4}=0$ or ${\alpha }_{1}=\cdots ={\alpha }_{L}=0$), the prediction performance drops significantly.

**Table 2:** MAE predicting CFC one hour into the future using the model in Eq. (2).

	*L* = 0	*L* = 1	*L* = 2	*L* = 3	*L* = 4	*L* = 5
With env.	0.144	0.050	0.049	0.049	0.049	0.049
Without env.		0.131	0.131	0.131	0.132	0.132

The third row refers to the model without the environmental variables (i.e. ${\beta }_{1}=\cdots ={\beta }_{4}=0$). *L* = 0 refers to a model without the autoregressive part (i.e. ${\alpha }_{1}=\cdots ={\alpha }_{L}=0$). If neither the autoregressive part or the environmental variables are included, the model will be reduced to a constant and is therefore not evaluated (bottom left cell in the Table).

## Usage Notes

The ECC dataset can be used to potentially improve the projection of future CFC. The dataset is mainly aimed for researchers and stakeholders working on applications where the projections of future CFC is important, such as agriculture and solar energy production.

We further think that the dataset can be interesting for ML researchers and professionals. The relation CFC and the environmental variables in the ECC dataset are complex with strong spatial and temporal dependencies and rapid changes, placing high demand on the ML methods and may open up for the development of new and better ML methods. We believe that DL-based methods can be useful, but we are only aware of one paper that has used the ECC dataset and DL to predict CFC^20^. Furthermore, using interpretation techniques, such as Explainable Artificial Intelligence^21^, new insight about cloud formation processes may be gained.

For the task of predicting CFC, metrics such as MAE or root mean squared error (RMSE) are appropriate. Regardless of the application, multiple metrics should always be reported for a full evaluation of an algorithm. For classification tasks (such as predicting clear sky or not), we recommend using standard classification metrics for either a binary or multi-class use case^22^. This includes metrics such as precision, recall, F1-score, and Matthews correlation coefficient.

## Data Availability

The Github repository (https://github.com/simula/european-cloud-cover.git) contains code to create the data set, load the data and to run the technical validation described in the previous section.
